# The role of anterior segment optical coherence tomography in the management of an intra-corneal foreign body

**DOI:** 10.1186/s40064-016-3242-x

**Published:** 2016-09-13

**Authors:** Ali Riza Cenk Celebi, Ayse Ebru Kilavuzoglu, Ugur Emrah Altiparmak, C. Banu Cosar, Abdullah Ozkiris

**Affiliations:** Department of Ophthalmology, Acibadem University School of Medicine, Turgut Ozal Boulevard, No: 16, 34303, Kucukcekmece, Istanbul Turkey

**Keywords:** Cornea, Foreign body removal, Anterior segment, Spectral domain optical coherence tomography, Cirrus SD-OCT

## Abstract

**Introduction:**

Corneal foreign bodies are reported to be the second most common type of ocular injury. Anterior segment optical coherence tomography (AS-OCT) is a valuable tool for the early diagnosis and monitoring the progress of treatment in cases of ocular trauma. Herein we aimed to report on a patient with an intra-corneal foreign body and the role of AS-OCT in management.

**Case presentation:**

A 34-year-old male presented with foreign body sensation in his left eye. Slit-lamp biomicroscopic examination revealed a peripherally located intrastromally embedded foreign body with a free anterior edge extending outwards from the cornea. It was not possible to visualize the foreign body’s entire route through the cornea because of localized corneal edema. AS-OCT showed shadowing of the corneal layers corresponding to the location of the corneal foreign body. A hyper-reflective lesion was observed close to the inside edge of the foreign body in the cornea, indicating that the foreign body had not completely penetrated the cornea. The foreign body was removed via the external route, as it had not completely penetrated the cornea. During the postoperative period the patient was asymptomatic, although the left eye’s cornea healed with scar tissue.

**Discussion and Evaluation:**

AS-OCT facilitates non-invasive rapid imaging of ocular tissue at va rious depths, thereby providing accurate assessment of foreign body characteristics.The location of an intracorneal foreign body and the status of the surrounding ocular structure dictate the optimal surgical technique to be employed.

**Conclusions:**

AS-OCT in the present case facilitated localization and determination of the size of a corneal foreign body. In addition, AS-OCT findings assisted in selection of the appropriate surgical intervention.

## Background

Ocular injury is the most common cause for emergency interventions in ophthalmology departments throughout the world, and is the second most common cause of visual loss (Nash and Margo 1998). Corneal foreign bodies are reported to be the second most common type of ocular injury, accounting for approximately 30.8 % of all eye injuries (McGwin and Owsley 2005). Some substances, such as sand and glass, are well tolerated in the cornea without precipitating a reaction. In such cases the inert foreign body can be left intrastromal while the patient undergoes close monitoring. If an inert foreign body in the cornea is in the visual axis and/or reduces visual acuity due to posterior astigmatism, prompt removal is indicated (Arora et al. 2015). Other substances, including metals (accounting for a major proportion of corneal foreign bodies) and such organic substances as vegetables and wood, are poorly tolerated by the cornea and cause localized edema, corneal opacification, inflammatory reaction, vascularization, and stromal necrosis, and must be promptly removed (Smolin and Thoft 1994).

Anterior segment optical coherence tomography (AS-OCT) is a valuable tool for the early diagnosis and monitoring the progress of treatment in cases of ocular trauma (Wylegala et al. 2009). AS-OCT uses low-coherence, near-infrared light to obtain detailed images of anterior segment structures, including cornea, at resolutions exceeding other available systems, including ultrasound biomicroscopy and conventional ocular ultrasonography (Simpson and Fonn 2008). OCT for anterior segment imaging, was first reported in 1994 (Izatt et al. 1994). Since then, significant technological advancements have resulted in the development of new-generation spectral domain OCT devices, including the Cirrus spectral domain optical coherence tomography (SD-OCT) device (Carl Zeiss Meditec, Dublin CA, USA), with a resolution of 5 µm and A-scan speed of 27.000 A-scan/s. Herein we report a patient with an intra-corneal foreign body and the role of AS-OCT in the management of this condition.

## Case presentation

A 34-year old male presented to our ophthalmology clinic with the sensation of a foreign body in his left eye. While performing his job as a blacksmith, he suddenly felt something hit his left eye. Ophthalmological examination showed visual acuity of 20/20 with pinhole correction in both eyes. Intraocular pressure was 11 mmHg in the left eye and 14 mmHg in the right eye. Both pupils were round, regular and equally reactive to light and accommodation.

**Fig. 1:**
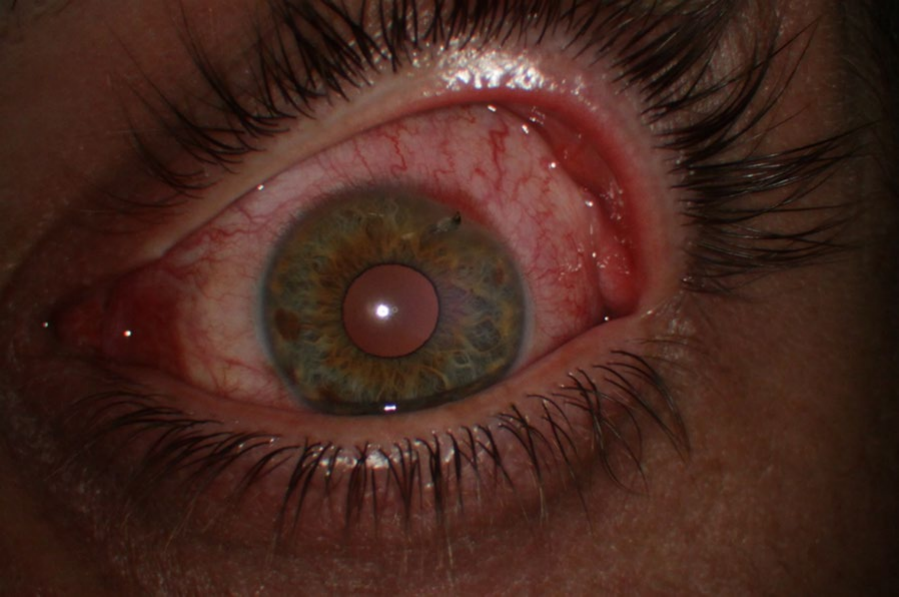
Preoperative slit-lamp biomicroscopy of the patient’s left eye shows the embedded corneal foreign body

Slit-lamp biomicroscopic examination of the left eye showed that there was an intrastromally embedded metallic foreign body with a free posterior edge extending outwards at the 1 o’ clock position. Significant eye redness was noted over the entire bulbar conjunctiva, which was more prominent in the limbus just behind to the entry site of the foreign body. An overlying corneal epithelial defect and localized surrounding corneal edema were noted at the point of foreign body entry. The foreign body had a free exit edge that was protruding outwards. It was not possible to visualize the entire route of the foreign body through the cornea because of localized surrounding corneal edema. The inside edge of the foreign body appeared to enter the anterior chamber via completely penetrating the cornea (Fig. 1). Gonioscopic examination was not performed due to the potential for the free posterior edge of the foreign body to be pushed into the anterior chamber. There was no apparent iris or lens damage, and no aqueous cells or aqueous flare were observed in the anterior chamber. Right eye slit lamp examination and fundoscopy findings in both eyes were unremarkable.

**Fig. 2:**
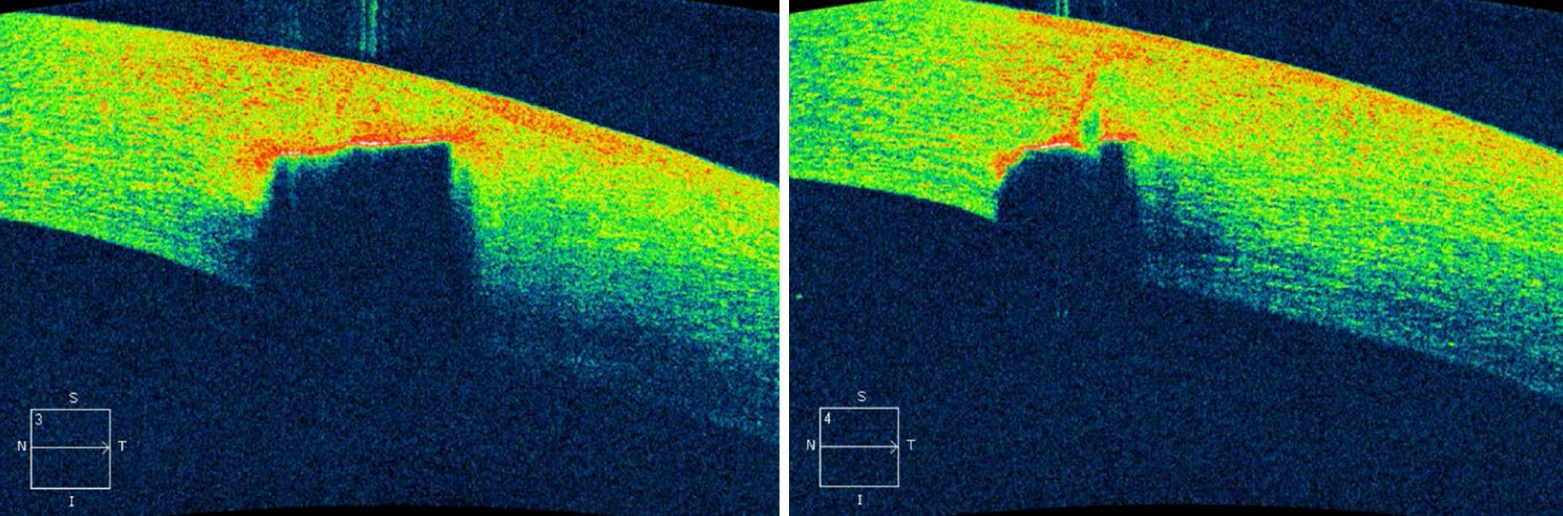
Pre-operative AS-OCT images of the patient’s left eye. AS-OCT image shows hyper-reflectivity and shadowing due to the corneal foreign body (*Left*). AS-OCT image shows that the corneal foreign body did not completely penetrate the cornea (*Right*)

AS-OCT was performed on the patient’s left eye using a Cirrus SD-OCT device in high-definition anterior segment 5-line raster scan mode. Details regarding scan protocol can be found elsewhere (Rodrigues et al. 2012). AS-OCT showed shadowing of the corneal layers that corresponded to the location of the foreign body (Fig. 2a). A hyper-reflective lesion was observed quite close to the inside edge of the cornea, indicating that the foreign body did not completely penetrate the cornea (Fig. 2b).

**Fig. 3:**
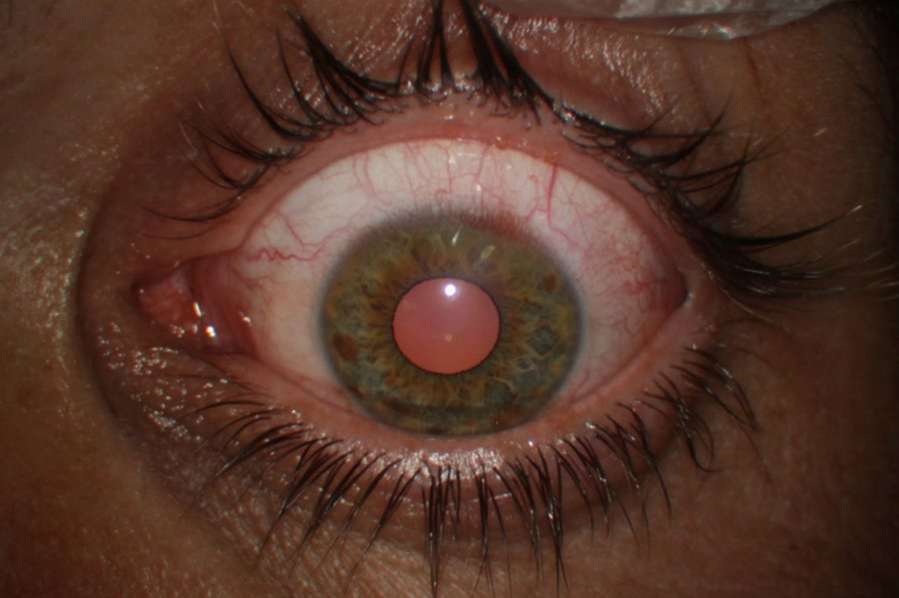
Postoperative slit-lamp biomicroscopy of the patient’s left eye shows the cornea beginning to heal with scar tissue formation

**Fig. 4:**
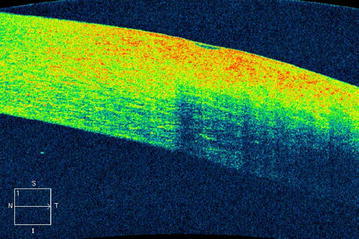
Postoperative AS-OCT image of the patient’s left eye shows hyper-reflectivity corresponding to corneal scar tissue

After explaining the treatment options and possible related consequences to the patient, he provided informed consent to undergo removal of the foreign body in an operating room under topical anesthesia. Based on the AS-OCT findings, the foreign body was removed via the external route, as it did not completely penetrate the cornea. Toothed grasping intraocular forceps was used to remove the foreign body via the same route as which it had entered. After removal of the foreign body, the Seidel test was administered and it did not show any fluorescein leakage. Based on the foreign body’s small size, site of entry, and its oblique route, post removal the entry site was not closed with sutures, but the eye was tightly covered with a bandage. Postoperative treatment was applied with lomefloxacin q.i.d and tobramycin ointment b.i.d. During the postoperative period the patient was asymptomatic and the left eye’s cornea healed with scar tissue (Fig. 3). AS-OCT performed 3 weeks after, showed hyper-reflectivity corresponding to the corneal scar tissue along the wound-healing line (Fig. 4).

The signed informed consent from the patient for the use of the unidentified personal and medical information and any accompanying images for the publication of this case report was obtained.

## Discussion

In clinical practice the cornea is routinely examined using slit-lamp biomicroscopy at magnification of 10×–25× (and up to 100×). Recent advances in ocular imaging include high-resolution techniques for visualizing cornea. Intrastromal corneal foreign bodies can be accurately viewed using the Cirrus SD-OCT. The advantages of SD-OCT are that it is a non-contact imaging system associated with minimal discomfort in trauma patients and that it can be used to obtain high-resolution cross sectional images of the anterior segment, including the cornea.

AS-OCT facilitates non-invasive rapid imaging of ocular tissue at various depths, thereby providing accurate assessment of foreign body characteristics. Reflectivity varies according to the type of foreign body. Foreign bodies of glass are well delineated on AS-OCT, with no internal reflectivity, whereas those of wood exhibit moderate internal reflectivity and those of metal exhibit high anterior reflectivity with shadowing (Jancevski and Foster 2010). The presented patient’s AS-OCT image showed high internal reflectivity with shadowing, indicating that it was a metal fragment. It is also important to discern the nature of corneal foreign bodies. Researcher examined the corneal wound healing process after injury due to a foreign body of iron, reporting that it resulted in a stromal scar and epithelial thickening (Maeda 2010). It was also noted a low-intensity shadow that they thought was a remnant of the iron foreign body that was not be detected via slit-lamp biomicroscopy. Foreign body composition determines the urgency of its removal and, metallic foreign bodies require urgent removal (Al-Ghadeer and Al-Assiri 2014).

Corneal foreign body imaging provides information regarding foreign body location, size and depth. In cases of ocular trauma AS-OCT can also identify unexpected lesions that are invisible or difficult to see via routine slit-lamp biomicroscopic examination (Wylegala et al. 2009). In the presented case preoperative AS-OCT confirmed that the foreign body did not completely penetrate the cornea, which was not clearly observed via slit-lamp biomicroscopy because of image overlap.

The high resolution of AS-OCT is ideal for evaluating the depth of corneal injury due to a foreign body. The location of an intracorneal foreign body and the status of the surrounding ocular structure dictate the optimal surgical technique to be employed (Hersh et al. 1995). Removal of a corneal foreign body via a lamellar corneal pocket was described by Au et al. (1996) in cases in which the entry wound caused by a foreign body had healed and epithelized. A lamellar dissection was extended centrally toward the corneal foreign body and the corneal wound was closed with a circumferential mattress suture of 10-0 nylon. In the presented case this technique was not indicated because the foreign body did not completely penetrate the cornea and there was a break in the corneal epithelial layer; instead, we simply grasped the corneal foreign body by its free posterior edge that was protruding outwards and removed it via its route of entry. The AS-OCT findings helped us choose the most appropriate technique for corneal foreign body removal.

AS-OCT provides vital details about Descemet’s membrane integrity and the point of entry of a foreign body, which can be utilized to plan surgical removal. When Descemet’s membrane was intact and the scar at the point of foreign body entry was known the foreign body was removed via the anterior route, whereas when Descemet’s membrane was breached and the foreign body point of entry was healed the foreign body was removed via the anterior chamber using an air tamponade. No sutures were required when removing a foreign body via the anterior chamber, which preventing any astigmatic effect. AS-OCT is a non-invasive method for rapid imaging of ocular tissue of various depths, which provides accurate localization of foreign bodies and the status of surrounding ocular structures, facilitating preoperative surgical planning. This can also prevent any intra or postoperative refractive surprises thus providing best possible outcomes (Al-Ghadeer and Al-Assiri 2014).

In some cases corneal thinning might be suspected after removal of a corneal foreign body. AS-OCT is extremely useful in such cases, as it facilitates quantitative assessment of remnant corneal thickness and indicates the risk of impending perforation. The presented patient was lucky in that that foreign body did not completely penetrate the cornea and there was no loss of tissue. In cases of corneal tissue loss cyanoacrylate glue and a bandage contact lens can be used if the defect is <2 mm (Vote and Elder 2000).

## Conclusion

The use of AS-OCT allowed to verify and even clarify clinical impression of the intra-corneal foreign bodies. This technology also served as a check on verification of depth and localization of foreign bodies. AS-OCT findings can also be used to aid in the selection of the most appropriate surgical intervention and to monitor the effects of treatment. The presented case highlights the benefits of AS-OCT in the management of intra-corneal foreign bodies, including informing the choice of method of foreign body removal.
